# Association Between Preoperative Cannabis Use and Increased Rate of Revision Surgery Following Spinal Fusion: A Systematic Review and Meta-Analysis

**DOI:** 10.7759/cureus.61828

**Published:** 2024-06-06

**Authors:** Rahul K Chaliparambil, Mehul Mittal, William Gibson, Christopher Ahuja, Nader S Dahdaleh, Najib El Tecle

**Affiliations:** 1 Neurological Surgery, Northwestern University Feinberg School of Medicine, Chicago, USA

**Keywords:** disc disorders, post-operative outcomes, pseudoarthrosis, spinal fusion, cannabis

## Abstract

The use of cannabis as a method of chronic pain relief has skyrocketed since its legalization in states across the United States. Clinicians currently have a limited scope regarding the effectiveness of marijuana on surgical procedures. This systematic review aims to determine the effect of current cannabis use on the rate of failure of spinal fusions and overall surgical outcomes. A systematic review was performed in accordance with the Preferred Reporting Items for Systematic Review and Meta-Analyses (PRISMA) statement. PubMed, Embase, and Scopus were searched, identifying studies assessing spinal fusion with reported preoperative cannabis use. Outcomes of interest included reoperation due to fusion failure or pseudoarthrosis with a follow-up time of at least six months. Subgroups of cervical fusions alone and lumbar fusions alone were also analyzed. Certainty in evidence and bias was assessed using the GRADE criteria and ROBINS-I tool (PROSPERO #CRD42023463548). Four studies met the inclusion criteria, with a total of 788 patients (188 in the cannabis user group and 600 in the non-user group). The rate of revision surgery among cannabis users was higher than that in non-users for all spinal fusions (RR: 3.58, 95% CI: 1.67 to 7.66, p = 0.001). For cervical fusions alone, there remained a higher rate of revision surgery for cannabis users compared to non-users (RR: 4.47, 95% CI: 1.93 to 10.36, p = 0.0005). For lumbar fusions alone, there was no difference in the rates of revision surgery between cannabis users and non-users (RR: 1.21, 95% CI: 0.28 to 7.73, p = 0.79). Cannabis use was shown to be associated with a higher rate of pseudoarthrosis revisions in spinal fusions on meta-analysis. On subgroup stratification by spine region, cannabis use remained associated with pseudoarthrosis revisions on cervical fusions alone but not lumbar fusions alone. Further research with larger, randomized studies is required to fully elucidate the relationship between cannabis use and fusion, both in general and by spinal region.

## Introduction and background

Reoperation due to fusion failure is a major concern following spinal fusion surgery. The rates of revision surgery can vary based on surgical approach and spinal region, but in national samples, rates have been shown to be around 5.3% in combined lumbar approach, 5.2% in posterior lumbar approach, 5.0% in anterior lumbar approach, 2.8% in posterior cervical approach, and 2.6% in anterior cervical approach [1]. Spinal fusion remains one of the most commonly performed orthopedic procedures, with more than 400,000 lumbar fusions and more than 127,500 anterior cervical discectomy and fusion surgeries performed annually in the United States as of 2013 [2,3]. With the demand for fusion procedures projected to increase dramatically in the coming decades, it is imperative to elucidate the factors that negatively impact the success of these procedures [4].

The use of cannabis as a recreational substance and for the management of axial back and radicular pains has dramatically increased in the past year [5-7]. The current understanding of the analgesic effects of cannabinoids stems from their ability to modulate nerve-ending depolarization and the stimulation of pain receptors by affecting neurotransmitter release in central and peripheral pain pathways [8,9]. However, cannabis abuse has been linked with perioperative neurologic and respiratory complications, sepsis, thromboembolism, post-operative length of stay, and unfavorable discharge disposition following elective spine surgery [10]. To the best of our knowledge, no systematic review of the existing literature evaluating the association between cannabis use and spinal fusion failure has been conducted. Based on this, this meta-analysis seeks to elucidate what is understood about this relationship and inform future research into this significant adverse event.

This article was previously presented as a meeting abstract at the 2024 AANS Annual Scientific Meeting on May 4, 2024.

## Review

Methods

Systematic Literature Review

A comprehensive search of PubMed, Embase, and Scopus databases was conducted along the Preferred Reporting Items for Systematic Review and Meta-Analyses (PRISMA) guidelines. The search strategy employed was ("cannabis" OR "marijuana" OR "weed" OR "cannabin**") AND ("spinal" OR "vertebral" OR "spine") AND ("surgery" OR "fusion" OR "spondylodesis" OR "spondylosyndesis" OR “arthrodesis” OR “pseudarthrosis”) (PROSPERO #CRD42023463548).

Study Selection

Title and abstract screening were performed by two blinded reviewers (R.K.C. and M.M.), and disputes were discussed until a consensus was achieved. Inclusion criteria were studies in English with adult patients (18 years of age or older) receiving spinal fusion surgery and comparing rates of revision surgery due to pseudoarthrosis between patients with a history of regular, preoperative cannabis use in any form versus patients without a history of preoperative cannabis use. The reference lists of included studies were additionally screened for more eligible publications. Studies of patients with spinal fusion in the case of malignancy, infection, and non-elective surgery were excluded. Studies in cadaveric models and animal models were also excluded. Finally, conference abstracts, existing reviews/meta-analyses, and studies utilizing public databases were also excluded.

Data Extraction

The variables extracted from eligible studies included sample sizes, age, gender, spine region operated, number of levels fused, surgical approach, rate of revision surgery, definition of bony fusion failure, and follow-up time. The primary outcome of interest was the rate of revision surgery. The Grading of Recommendations Assessment, Development, and Evaluation (GRADE) framework was utilized to assess quality [11]. The Risk of Bias of Nonrandomized Studies of Interventions tool (ROBINS-I) was utilized to determine the risk of bias [12]. The corresponding authors of the included studies were contacted in the case of missing data.

Statistical Analysis

Meta-analysis was performed to compare the outcomes of spinal fusion patients with preoperative cannabis use compared to those without preoperative cannabis use. Forest plot generation and statistical analysis of the outcomes of interest were conducted in Review Manager 5.4 (Cochrane Collaboration, Copenhagen, Denmark). Generated forest plots depicted the corresponding risk ratio (RR) and 95% confidence interval (CI) for each outcome. The random-effects (DerSimonian and Laird) method for meta-analysis was utilized. Heterogeneity between studies was assessed using the I2 statistic. A p-value of < 0.05 was used as the statistically significant threshold for all analyses.

Results

**Figure 1:**
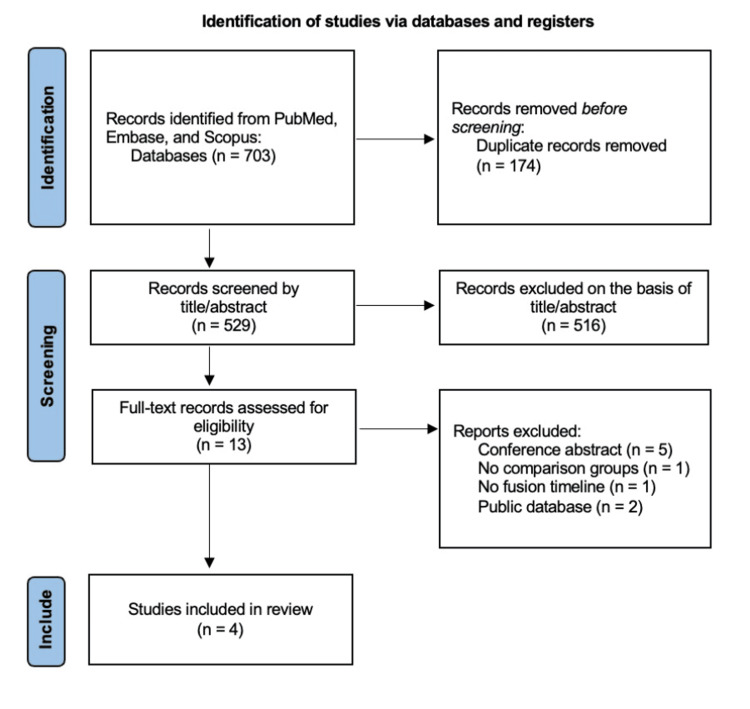
PRISMA flowchart of the included studies PRISMA, Preferred Reporting Items for Systematic Review and Meta-Analyses

The search strategy yielded 703 articles, of which 174 were duplicates. Of the 529 unique articles, 516 were excluded on the basis of title and abstract, and nine were further excluded on full-text review, leaving four studies for final analysis [13-16] (Figure 1). All studies were retrospective, comparative, single-center studies. A total of 788 patients were included in analysis, with 600 patients in the non-user group and 188 patients in the pre-operative cannabis user group (Table 1).

**Table 1:** Characteristics of included studies Abbreviations: BMI, body mass index; GRADE, Grading of Recommendations Assessment, Development, and Evaluation; PLIF, posterior lumbar interbody fusion; ROBINS-I, Risk of Bias of Nonrandomized Studies of Interventions tool; SD, standard deviation; TLIF, transforaminal lumbar interbody fusion

Study	Year	Pre-operative cannabis users, n (%)	Non-users, n (%)	Age, mean (SD)	Male, n (%)	BMI, mean (SD)	Spine region	Number of levels fused	Surgical approach	Definition of bony fusion failure / pseudoarthrosis	Follow-up time for revision surgery, years	Grade	ROBINS-I
Lambrechts et al. [14]	2022	60 (25.0)	180 (75.0)	54.2 (10.7) for cannabis users, 54.3 (11.6) for non-users	32 (53.3) for cannabis users, 95 (52.8) for non-users	28.2 (5.76) for cannabis users, 28.5 (5.23) for non-users	Cervical	1, 2, 3, or 4	Anterior	Computed tomography on symptomatic patients	3	Low	Moderate
Razzouk et al. [16]	2022	13 (7.0)	174 (93.0)	52.5 (N/A) for cannabis users, 56.3 (N/A) for non-users	8 (61.5) for cannabis users, 74 (42.5) for non-users	27.7 (N/A) for cannabis users, 30 (N/A) for non-users	Cervical	1 or 2	Anterior	Recorded pseudoarthrosis	2	Low	Serious
D’Antonio et al. [13]	2022	65 (20.0)	259 (80.0)	57.3 (N/A) for cannabis users, 56.1 (N/A) for non-users	32 (49.2) for cannabis users, 95 (49.0) for non-users	30.6 (5.86) for cannabis users, 30.9 (6.07) for non-users	Thoracolumbar	1 or 2	TLIF, PDLF, circumferential	Computed tomography on symptomatic patients	3	Low	Moderate
Jakoi et al. [15]	2020	36 (35.3)	66 (64.7)	52.6 (N/A) for cannabis users, 61.8 (N/A) for non-users	27 (54.0) for cannabis users, 25 (48.1) for non-users	37.2 (7.7) for cannabis users, 38.2 (8.2) for non-users	Thoracolumbar	1 or 2	TLIF	Radiologically confirmed via the Lenke grading system, Cobb angle difference <58, and screw haloing or pullout	1	Low	Moderate

**Figure 2:**
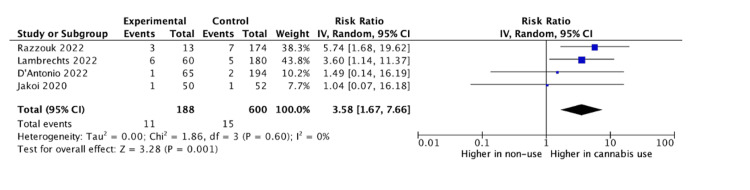
All fusions Razzouk et al. [16], Lambrechts et al. [14], D'Antonio et al. [13], Jakoi et al. [15]

**Figure 3:**
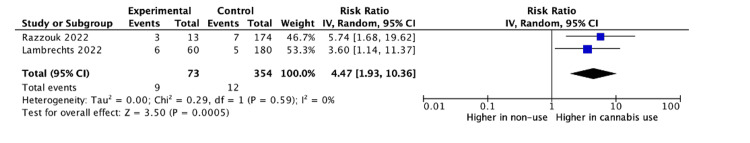
Cervical only Razzouk et al. [16], Lambrechts et al. [14]

**Figure 4:**
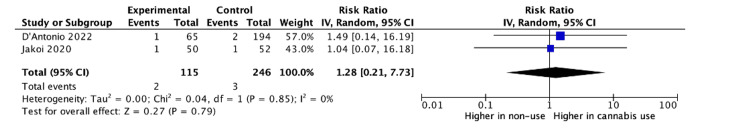
Lumbar only D'Antonio et al. [13], Jakoi et al. [15]

Baseline differences between cannabis users and non-users across all studies were assessed via a meta-analysis. Razzouk et al. did not report on the variance for age or body mass index (BMI), and thus these variables excluded from baseline analysis [16]. There was no significant difference between groups in male sex (odds ratio (OR): 1.17, 95% CI: 0.83 to 1.65, p = 0.37), age (mean difference (MD): -2.35, 95% CI: -7.91 to 3.20, p = 0.41), or BMI (MD: - 0.39, 95% CI: -1.48 to 0.71, p = 0.49). The rate of revision surgery among cannabis users was higher than in non-users for all spinal fusions (RR: 3.58, 95% CI: 1.67 to 7.66, p = 0.001) (Figure 2). For cervical fusions alone, there remained a higher rate of revision surgery for cannabis users compared to non-users (RR: 4.47, 95% CI: 1.93 to 10.36, p = 0.0005) (Figure 3). For lumbar fusions alone, there was no difference in the rates of revision surgery between cannabis users and non-users (RR: 1.21, 95% CI: 0.28 to 7.73, p = 0.79) (Figure 4).

Discussion

Revision spine surgeries have been associated with higher rates of complications and 30-day readmission or reoperation when compared to primary surgery, making an understanding of the risk factors associated with bony fusion failure following spine surgery vitally important [17]. Our study identified a statistically significant difference in revision surgery rates between cannabis users and non-users on meta-analysis. On subgroup analysis, this difference was maintained in cervical fusions alone but not in lumbar fusions alone.

Cannabis use in the long-term management of spinal surgery is growing in popularity with ongoing legalization in the United States [5-7]. With preoperative prescription opioid use being linked to negative surgical and functional outcomes, including postoperative opioid use, hospitalization duration, healthcare costs, and risk of surgical revision, there remains an urgent need to find solutions to combat routine opioid use [18]. A major benefit of cannabis incorporation into pain management is the ability to reduce the degree of prescription opioid use in preoperative and postoperative periods [19]. Research in this area can help better understand if cannabinoids have a role in combating opioid dependence without significantly impacting surgical outcomes.

Common causes for pseudoarthrosis seen in the literature include, but are not limited to, smoking status, NSAID use, bone graft material, location, and fusion construct [20]. The mechanism for fusion failure following cannabis use is poorly understood but has been hypothesized by the literature. Cannabinoids have been shown to regulate bone metabolism through the endocannabinoid system and have been associated with a protective effect against the development of osteoarthritis and osteoporosis in rodent in vivo animal models [21]. However, heavy cannabis use has been shown to be associated with low bone mineral density and an increased risk of fractures, mediated through a low BMI [22]. Several studies have shown a high association between cannabis use and the use of other substances, including opioids and nicotine [23-25]. Both opioid and nicotine abuse have been well understood to affect fusion rate but were not routinely collected in our included studies.

The basis of the relationship between the region of spine surgery and the rate of revision surgery is unclear. While the included studies in this meta-analysis for lumbar operations showed no association with cannabis use and fusion failure, some multi-center database studies have shown higher rates of pseudoarthrosis among cannabis users following lumbar fusion [26,27]. In general, the findings in the literature remain contradictory, with some studies even suggesting a protective effect against-short term complications of spinal surgery among cannabis users [28]. Lumbar fusions have broadly shown to be associated with higher rates of reoperation when compared to cervical fusions, potentially attributable to higher biomechanical forces at lumbar joints including higher weight and more local pressure [29,30]. This may suggest that fusion failure due to biomechanics supersedes the effects of routine cannabis use in the case of lumbar fusions alone, although this relationship requires further investigation in future prospective studies.

This study comes with several limitations inherent to the format. The small sample size of papers and the retrospective design of the included studies require a cautious interpretation of the conclusions. Male sex, age, and BMI were the only baseline variables that were able to be collected and compared across studies, leaving several covariates uncontrolled for, including smoking status and opioid use. Marijuana has numerous forms of consumption with varying ratios of tetrahydrocannabinol (THC), cannabidiol (CBD), and cannabigerol (CBG) across products that can modulate its psychotropic and analgesic effects [30,31]. The method of use or relative ratio of THC, CBD, and CBG in used products was not reported in any of the retrospective studies included and should be assessed as a predictor of fusion outcomes in future work. There was a discrepancy in follow-up length for revision surgery between the included studies as well, with one study having a one-year follow-up, one study having a two-year follow-up, and two studies having a three-year follow-up. In a large national cohort study, the rate of revision operations within two years for spinal fusion was 1.7%, while for three-year follow-up, the rate increased to 4.5% [1]. As more studies in this area are being conducted, controlling for length of follow-up can be important in understanding these relationships. In addition, there is a lack of information regarding important surgical cofounders including if concurrent laminectomies were performed, the reason for the operation, and the type of graft material. Future studies should make a point to include these variables to improve the clinical utility of recommendations. Finally, these surgeries were performed by many different surgeons over a range of clinical centers in the United States, which may have led to the noted outcomes due to factors such as surgeon experience, surgical indications, and postoperative management.

## Conclusions

The results of this systematic review and meta-analysis bring to attention concerns for routine cannabis use in the setting of spinal fusion surgery. Cannabis use was shown to be associated with a higher rate of pseudoarthrosis revisions in spinal fusions on meta-analysis. On subgroup stratification by spine region, cannabis use remained associated with pseudoarthrosis revisions on cervical fusions alone but not lumbar fusions alone. The results of this study provide impetus for the use of larger, multi-institutional or registry studies to fully elucidate the relationship between cannabis use and fusion, both in general and by spinal region.
